# Simultaneous isotopic analysis of fission product Sr, Mo, and Ru in spent nuclear fuel particles by resonance ionization mass spectrometry

**DOI:** 10.1038/s41598-023-32203-5

**Published:** 2023-03-30

**Authors:** Michael R. Savina, Brett H. Isselhardt, Danielle Z. Shulaker, Martin Robel, Andrew J. Conant, Brian J. Ade

**Affiliations:** 1 Lawrence Livermore National Laboratory, Nuclear and Chemical Sciences Division, Livermore, CA USA; 2 Oak Ridge National Laboratory, Material Security and Counterproliferation, Nuclear Nonproliferation Division, Oak Ridge, TN USA; 3 Oak Ridge National Laboratory, Research and Test Reactor Physics Group, Nuclear Energy and Fuel Cycle Division, Oak Ridge, TN USA

**Keywords:** Mass spectrometry, Nuclear chemistry, Nuclear fuel

## Abstract

Fission product Sr, Mo, and Ru isotopes in six 10-μm particles of spent fuel from a pressurized water reactor were analyzed by resonance ionization mass spectrometry (RIMS) and evaluated for utility in nuclear material characterization. Previous measurements on these same samples showed widely varying U, Pu, and Am isotopic compositions owing to the samples’ differing irradiation environments within the reactor. This is also seen in Mo and Ru isotopes, which have the added complication of exsolution from the UO_2_ fuel matrix. This variability is a hindrance to interpreting data from a collection of particles with incomplete provenance since it is not always possible to assign particles to the same batch of fuel based on isotopic analyses alone. In contrast, the measured ^90^Sr/^88^Sr ratios were indistinguishable across all samples. Strontium isotopic analysis can therefore be used to connect samples with otherwise disparate isotopic compositions, allowing them to be grouped appropriately for interpretation. Strontium isotopic analysis also provides a robust chronometer for determining the time since fuel irradiation. Because of the very high sensitivity of RIMS, only a small fraction of material in each of the 10 μm samples was consumed, leaving the vast majority still available for other analyses.

## Introduction

One of the goals of nuclear material security is to infer the histories of nuclear facilities in order to ascertain the unauthorized production of nuclear materials^1–3^. This may involve the analysis of spent fuel from reactors, often in the form of particles. Spent fuel particulate analysis presents difficulties. The elemental and isotopic composition of spent fuel varies widely depending upon the local irradiation conditions within the reactor core. For example fuel elements near the center of the core experience greater neutron flux than those near the top or bottom or the outer edges^4^. This results in greater production of fission products, and also in greater transmutation of those species due to neutron capture. In addition, elemental and isotopic compositions can change significantly within a few hundred micrometers of the edge of an individual fuel pellet due to the skin effect, in which epithermal neutrons are strongly captured by ^238^U, resulting in copious production of Pu^5–8^. This in turn results in changes in the fission product distribution at the pellet edge because fission products of ^239^Pu have a different elemental and isotopic distribution than those of ^235^U.

Samples of unknown provenance could come from anywhere inside the reactor or from different reactor cycles (i.e., from different fueling/defueling events). Therefore, a collection of particles may be biased since a representative sampling is unlikely. This confounds efforts to reconstruct the core-averaged irradiation history of a batch of fuel from a given reactor cycle (i.e., the source term), and thus predict the amounts and isotopic compositions of various byproducts such as plutonium produced by the reactor. The amount of material in any given particle is limited, so analytical methods with high sample utilization are required. Because of these challenges in deriving source term information from a collection of discrete particles, techniques that yield information on many analytes simultaneously in small particles are of great value.

Given that all the particles in a collection may not come from the same source term and thus may have experienced different irradiation histories, the age of a particle of spent fuel, expressed as the time since last irradiation in a reactor, can be an important piece of information. Particle ages sort samples into coherent batches for interpretation despite the wide variation in the isotopic compositions seen in samples from the same reactor cycle or even the same fuel pellet. Radiochronometric pairs such as ^241^Pu–^241^Am give information on the age of spent fuel, however measuring the age via ^241^Pu decay even on relatively large particles such as those investigated here leads to high uncertainties in sample ages^8^. In this work we show that Sr isotopes are well suited for this purpose. Stable ^88^Sr and radioactive ^90^Sr (t_½_ = 28.8 year) have high fission yields, are easily measured by RIMS, and can yield sample ages.

Molybdenum and Ru are strongly produced by fission, but their isotopic compositions are more difficult to interpret with respect to their production in reactors. The isotopic composition of fission product Ru has been proposed as a burnup indicator^9^. Burnup is a measure of the energy generated per unit mass of fuel, and is a proxy for the total number of fissions from all fissile isotopes. In addition, Ru may be a proxy for the fraction of total fissions due to ^239^Pu (*F*_*Pu*_), which increases with burnup and is higher at the pellet edge than in the center^10^. However, Ru and Mo undergo neutron capture in the reactor after they are produced and, together with Tc, Rh and Pd, are known to exsolve from solid UO_2_ to form dissolution-resistant metallic particles up to ~ 1 μm in diameter known as epsilon particles^9,11–14^, which can complicate interpretation.

While several techniques are in general use for spent fuel analysis, we concentrate on mass spectrometry in this discussion. (For an overview of mass spectrometric analysis in nuclear forensics see Ref.^15^). Mass spectrometric analysis of actinides and fission products in spent nuclear fuel have been used to ascertain reactor operating parameters such as burnup and residence time^8,16–18^. Fission product Sr has been well studied mainly in environmental analyses. Strontium-90 has been found in the environment due to the Fukushima^19,20^ and Chernobyl^21^ nuclear power plant releases, as well as from nuclear weapons testing^22^. Mass spectrometric detection of ^90^Sr must avoid isobaric ^90^Zr, and there are several reviews of ^90^Sr mass spectrometry in the literature^23–25^. The most common methods rely on dissolution and chromatographic purification of Sr to remove ^90^Zr, followed by analysis by Thermal Ionization Mass Spectrometry (TIMS) or Inductively-Coupled Mass Spectrometry (ICP-MS). Recently, the reaction of Zr with O_2_ in the collision cell of an ICP-MS has been shown to be effective in Sr isotopic analysis. This method requires dissolution but not chromatography and thus saves an analytical step. The preferential oxidation of Zr over Sr can also be exploited in a simple laser ionization mass spectrometer to perform Sr analysis without interference^26^.

Resonance ionization mass spectrometry (RIMS) has several advantages over other mass spectrometric techniques for this application. First, RIMS has demonstrated very high sensitivity compared to other techniques^16,27–30^, making it well suited to microprobe analysis of particles. Second, RIMS has the ability to discriminate against isobars by selectively ionizing only the elements of interest while leaving isobars of other elements as undetected neutral atoms^31^ thus obviating the need to dissolve and separate the material, which is difficult to do on small particles. Finally, when isobars are encountered during simultaneous analysis of multiple elements, delayed ionization of one element can be used to resolve—rather than discriminate against—isobars^6,32^.

RIMS has long been used to measure Sr, Mo, and Ru isotopic compositions, including for example to determine isotope shifts^33,34^, to measure isotopic compositions in stardust grains^35–40^, and to develop RIMS ion imaging^41^. RIMS methods have also been developed to measure ^90^Sr with high abundance sensitivity^42,43^, and to perform geochronology using ^87^Rb-^87^Sr^44,45^.

In the present work we develop and demonstrate a RIMS method to determine Sr, Mo and Ru isotopic compositions simultaneously. The samples were six spent UO_2_ nuclear fuel cubes, 10 μm on a side, which were previously analyzed for U, Pu, and Am isotopic compositions by RIMS^6^. Sample consumption from both of these analyses was extremely low, such that nearly all of the original material is left for further analysis. We compare our spatially resolved RIMS results with previous bulk analyses of Mo and Ru in the same spent fuel samples, and we use our Sr results to determine the ages of the samples. We find that spatially resolved Mo and Ru isotopic compositions show wide variations from sample to sample and do not in general correlate with bulk measurements on the same fuel pellets, indicating that variations on the micrometer scale, likely due to diffusion and exsolution, are non-negligible. In contrast, Sr analysis shows a consistent ^90^Sr/^88^Sr ratio for all six cubes, and therefore can group samples with widely disparate actinide and fission product isotopic compositions by source term. Age determination based on ^90^Sr decay yields accurate ages with a precision of 0.6 years.

## Methods

### Samples

**Figure 1:**
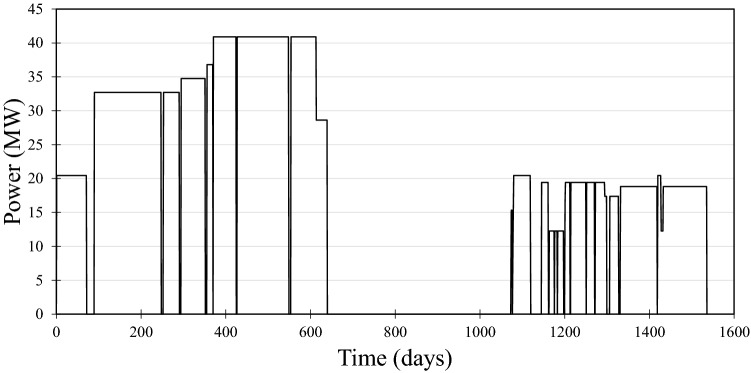
A simplified history of the power experienced by the fuel pellets in this study during the two irradiation periods of the reactor.

Powdered SrTiO_3_, Mo_2_C, and Ru metal were used as isotopic standards. The spent fuel samples were the same 10 μm UO_2_ cubes we previously analyzed for U, Pu, and Am isotopic composition in an earlier study^6^, of which the vast majority of material still remained. The samples came from the Belgian Reactor No. 3 (BR3) pressurized water reactor (PWR). This fuel had an initial enrichment of 8.26% and was discharged in September of 1980. It underwent two separate irradiations, from July 1976 to April 1978, and from June 1979 to September 1980. Thus, there were operation periods of 1.75 and 1.26 years, with a 1.2-year shutdown period in between during which the fuel sat dormant in the reactor. The reactor power level varied during operation but was between 35 and 41 MW/t most of the time. While the reactor power levels for the first and second irradiations were roughly the same, the fuel rod from which these samples were cut was moved from near the center of the core during the first irradiation period to near the edge for the second. As a result, the power experienced by these samples during the second irradiation period was approximately half of that during the first period. Figure 1 is a simplified power history experienced by the fuel in this study.

Sample cutting and mounting is described in detail elsewhere^6,8^. Two sets of three 10 μm cubes from two different fuel pellets (originally 8 cm in diameter) within the same fuel rod were cut using a focused ion beam and mounted on copper holders. One fuel pellet was halfway between the center and the top of the rod while the other was from near the rod center (axial sampling positions 2 and 3 in Ref.^8^). The pellet-average burnups were estimated to be 39 and 54 GWd/t based on their gamma activities. Cubes were cut from three radial positions within each pellet: two within 200 μm of the edge and one near the center.

### Measurements

All RIMS measurements were made on the LION instrument at Lawrence Livermore National Laboratory. The RIMS technique and the LION instrument have been described in detail elsewhere^29,46,47^. A Nd:YAG laser (1064 nm, 7 ns full width at half-maximum, 1000 Hz) focused to a 1–2 µm spot volatilized material from the samples. After a variable delay, neutral gas-phase atoms were resonantly ionized with pulses from tunable Ti:Sapphire lasers aligned colinearly ~ 1 mm above the sample surface. The photoions were then accelerated into a time-of-flight mass spectrometer.

Previously developed two-color/two-photon resonance ionization spectroscopy (RIS) schemes were used for Sr^36^, Mo^35^, and Ru^48^. The laser parameters are shown in Table 1. To resolve the Mo and Ru isobars at *m/z* 100, the ionization of Ru was delayed with respect to Sr and Mo, in a manner previously described to separate Pu isobars from U and Am^6^.

**Table 1:** RIS laser parameters.

Laser^a^	Wavelength (nm)	Power (W)	Diameter^b,c^ (mm)	Pulse width^b^ (ns)	Irradiance (MW/cm^2^)
Sr (I)	460.862	0.05	1.5	38	0.074
Sr (II)	405.214	0.26	1.75	7	1.5
Mo (I)	313.350	0.015	1.25	8	0.15
Mo (II)	388.337	0.51	1.8	21	0.95
Ru (I)	287.583	0.04	1.25	7	0.47
Ru (II)	403.983	0.64	1.25	17	3.1

^a^The pulse repetition rate for all lasers was 1 kHz. Roman numerals refer to the step in the ionization process.

^b^Full width at half maximum. ^c^Average of major and minor axes; ellipticities ranged from 1.0 to 1.3.

Only ^88^Sr and ^90^Sr are produced by fission, but we did not measure a standard containing ^90^Sr. Table 2 shows the measured stable Sr isotope ratios in the standard. Our RIMS measurements on natural Sr showed isotopic fractionations for the stable isotopes consistent with zero within 1σ (see supplementary information for a derivation of analytical uncertainties). RIMS fractionations in even-even isotope ratio measurements arise from transition isotope shifts that are significant compared to the bandwidth of the lasers. The data of Table 2 shows no significant fractionations. Estimates of the ^90^Sr transition isotope shifts relative to ^88^Sr are consistently much less than 1 GHz^49,50^. In particular, Lorenzen^34^ showed that the transition isotope shift for the 5*s*^2^(^1^S_0_) → 5*s*5*p*(^1^P_1_) transition used in this work is mass-dependent with a proportionality constant of ~ 0.06 GHz/amu. The ^88^Sr-^90^Sr pair is separated by 2 amu, so we expect an isotope shift of ~ 0.12 GHz. This is well within the 10 GHz bandwidth typical of our lasers and therefore we do not expect isotopic fractionation in the ^90^Sr/^88^Sr ratio due to the lasers.

**Table 2:** Sr isotopic standard measurements.

	^84^Sr/^86^Sr	^87^Sr/^86^Sr	^88^Sr/^86^Sr
Measured Ratio	0.0576(13)	0.7132(60)	8.336(48)
Known Ratio	0.0568	0.7099	8.375
Deviation^a^	1.5 ± 2.3%	0.5 ± 0.8%	− 0.5 ± 0.6%

Uncertainties in the isotope ratios are 1σ. (see Supplemental Material for calculation of uncertainty).

^a^Deviation = 100 × (R_measured_/R_known_−1).

In addition to the cubes analyzed here, one-gram portions of the fuel pellets were previously dissolved and analyzed by ICP-MS at Idaho National Laboratory^51^. The sampling favored the rims of the pellets but included significant portions of the center as well. We therefore expect isotope ratios measured this way to lie on a mixing line between center and edge compositions. Since no chemical separations were done prior to ICP-MS, not all isotopes were analyzed due to isobaric interference. We hereafter refer to these measurements as bulk analysis.

### Models

ORIGEN-ARP Version 6.1 was used to model an analog to the BR3 reactor to predict approximate spent fuel composition under a variety of simplified operating histories. The Westinghouse 17 × 17 model was used with a fuel enrichment of 6%, which is the highest available in the ARP libraries. The detailed operating history was smoothed to produce a core average power of 32 MW, which was then scaled. For this analysis, we scaled the power of the core model to simulate the fuel from which these samples originated. The shutdown period was included, and the power was scaled by ½ for second irradiation period.

A pin cell model in the NEWT/TRITON sequence in the SCALE code system^52^ was developed based on parameters from previous studies^4,53^ to examine the radial dependence of isotope concentrations. The model was a pin cell separated into 99 radial regions, with smaller thickness cells near the radial edge of the pin. From the outer edge, the model had 25 cell regions with thicknesses of 10 µm, followed by 60 cell regions with thicknesses of 20 µm, with the remaining cell regions having progressively coarser thicknesses to the pin center. The pin cell was depleted with twenty constant-power time steps using the same power profile as the ORIGEN model. Output concentration and flux library files were generated for subsequent ORIGEN decay analysis to account for the time between fuel discharge and RIMS measurements.

In addition to these rigorous models which calculate abundances of all isotopes, we developed a simple method to estimate the ^90^Sr/^88^Sr ratio at discharge which considers only ^88^Sr and ^90^Sr produced by thermal neutron fission of ^235^U and ^239^Pu in the reactor, with continuous decay of ^90^Sr. Hereafter we refer to this as the TF (thermal fission) model. We used thermal fission yields from ENDF/B-VIII^54^. Neutron capture on Sr was neglected, as the relevant cross sections are only a few millibarns. The model was run for the known operation period of the reactor with a time step of one day using two different power profiles: that of Fig. 1, as well as the simpler profile used in the ORIGEN and pin cell models.

## Results and discussion

### RIMS analysis

**Figure 2:**
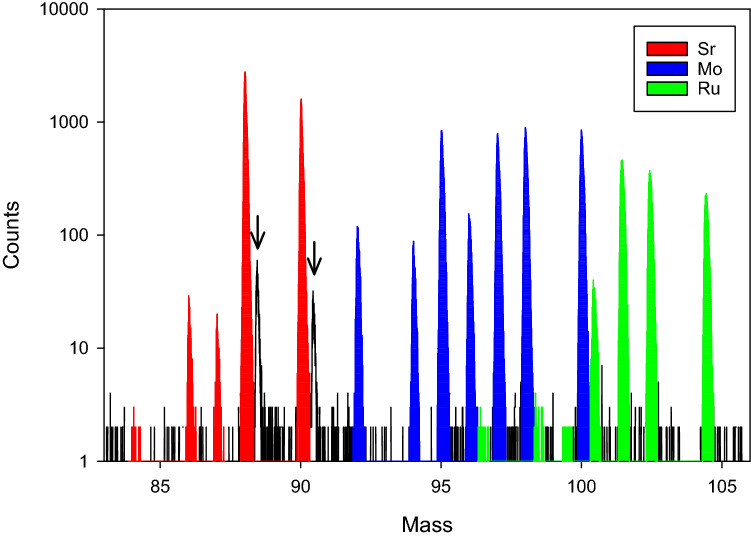
RIMS spectrum showing Sr (red), Mo (blue) and Ru (green) isotopes in spent nuclear fuel. The arrows indicate Sr ionized non-resonantly by the Ru lasers, which were delayed relative to the Sr and Mo lasers (see text).

Figure 2 shows a RIMS spectrum from one of the spent fuel samples obtained with the three-element laser setup described in the Experimental section. The delayed ionization of Ru with respect to Mo and Sr results in an apparent mass shift of 0.4 amu in the time-of-flight spectrum. Fission product ^95,97,98,100^Mo and ^101,102,104^Ru peaks are observed, as well as ^100^Ru formed by neutron capture on ^99^Tc followed by β^−^ decay. Non-fission Mo peaks are seen at *m/z* 92, 94, and 96, and are presumed to be due to Mo contamination, but no non-fission Ru is observed (e.g., ^99^Ru). Strontium peaks at *m/z* 84, 86, 87, 88 and 90 are all visible, though only ^88^Sr and ^90^Sr are produced by fission. In addition, there are peaks at 88.4 and 90.4 amu (indicated with arrows in Fig. 2) attributable to off-resonant ionization of ^88^Sr and ^90^Sr by the time-delayed Ru ionization lasers. These peaks represent ~ 1% of the resonant Sr signal and are baseline resolved from the resonant Sr peaks.

Notably absent in the spectrum of Fig. 2 is Zr, which would be observable at *m/z* 91 (stable ^91^Zr) and *m/z* 93 (^93^Zr; t_½_ = 1.5 × 10^6^ year). ORIGEN and pin cell models predict copious production of these two fission products, with ^91^Zr/^88^Sr and ^93^Zr/^88^Sr ratios of 1.6 and 2.0 respectively. This is borne out by the bulk analysis, which found ratios of 1.85 and 2.11, respectively. Given the age of the fuel we expect ^90^Zr/^90^Sr = 1.04 from β^−^ decay of ^90^Sr, which means that both ^91^Zr and ^93^Zr are more abundant than ^88^Sr in this material. The ratio of *m/z* 88 to *m/z* 93 in the spectrum is > 15,000:1. This implies a discrimination factor of > 30,000 for Sr over Zr. We conclude that there is no significant interference from ^90^Zr on the ^90^Sr peak.

**Figure 3:**
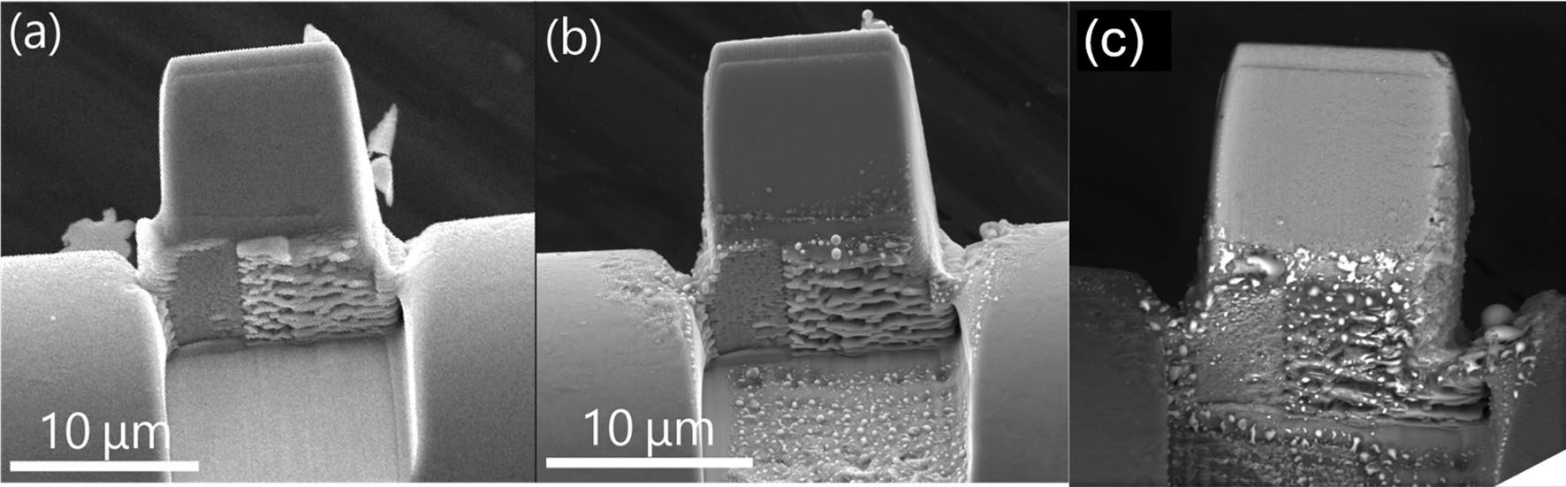
Electron micrographs of one of the sample cubes as received (**a**), after U, Pu, and Am isotopic analysis (**b**), and after further analysis for Mo, Ru, and Sr isotopic composition (**c**).

As noted in the Introduction, RIMS is an extremely sensitive mass spectrometric technique, and simultaneous analysis of multiple elements makes it even more so. Figure 3 shows electron micrographs of one of the samples before and after two RIMS analysis sessions that measured first U, Pu, and Am, and then Mo, Ru, and Sr isotopic compositions. The mass of the cube is ~ 10 ng, and the concentrations of Sr, Mo and Ru as measured by bulk analysis are 871(1), 3596(8), and 2340(8) μg/g respectively. The figure shows the face of the cube after 2.2 × 10^6^ laser shots for the previous actinide analysis and an additional 1.6 × 10^6^ shots for the present Mo, Ru, and Sr analysis (the repetition rate was 1 kHz). Some melting of the Pt weld and Cu holder and some erosion of the UO_2_ at the right edge of the cube is evident, however there are no visible laser pits and we cannot determine from these images how much material was consumed. It is evident however that very little material was consumed. For reference, a 10 μm cube contains 9, 26, and 23 pg of Sr, Mo, and Ru respectively.

We used the ORIGEN model to estimate the abundances of minor Sr and Mo isotopes to correct for the natural Sr and Mo contamination observed in the RIMS spectra. ORIGEN provides elemental and isotopic composition estimates for bulk fuel. In this case it predicts essentially zero production of ^92,94^Mo so these two isotopes were used to correct for Mo contamination, which was present in every sample. The amount of contamination was highly variable from sample to sample and accounted for anywhere from 1 to 43% of the total Mo observed. Like U, Mo forms a gaseous hexafluoride and can be carried along during the enrichment process, however the amount observed here is far too high to have been introduced via this route. Analysis of a low-enriched U standard (CRM-125A) indicates ~ 1–10 ppm of Mo^55^, while analyses of other low-enriched U materials show 10–100 pm^56^. Comparing ORIGEN predictions to the RIMS measurements, we estimate that up to ~ 1300 ppm of contaminant Mo is present in our samples, which is much more than can be accounted for by initial Mo content. Further, Mo present in the virgin fuel would lead to a constant Mo content in all samples, which was not observed here. The large sample-to-sample variability indicates that Mo was introduced during sample preparation and handling.

Weak production of ^84,86,87^Sr is expected in a PWR, but the amounts noted in two of our samples were higher than could be accounted for by irradiation. For example, ORIGEN predicts ^87^Sr/^88^Sr < 10^–6^, yet a robust ^87^Sr peak is noted in the spectrum of Fig. 2. Using the ^87^Sr abundance to correct for non-fission ^88^Sr contamination changes the ^90^Sr/^88^Sr ratio of this sample by 6.3%. The contamination correction on one other cube changed the ratio by 2.3%; the corrections on the four others were all less than 0.1%.

### Mo and Ru isotopic compositions

**Figure 4:**
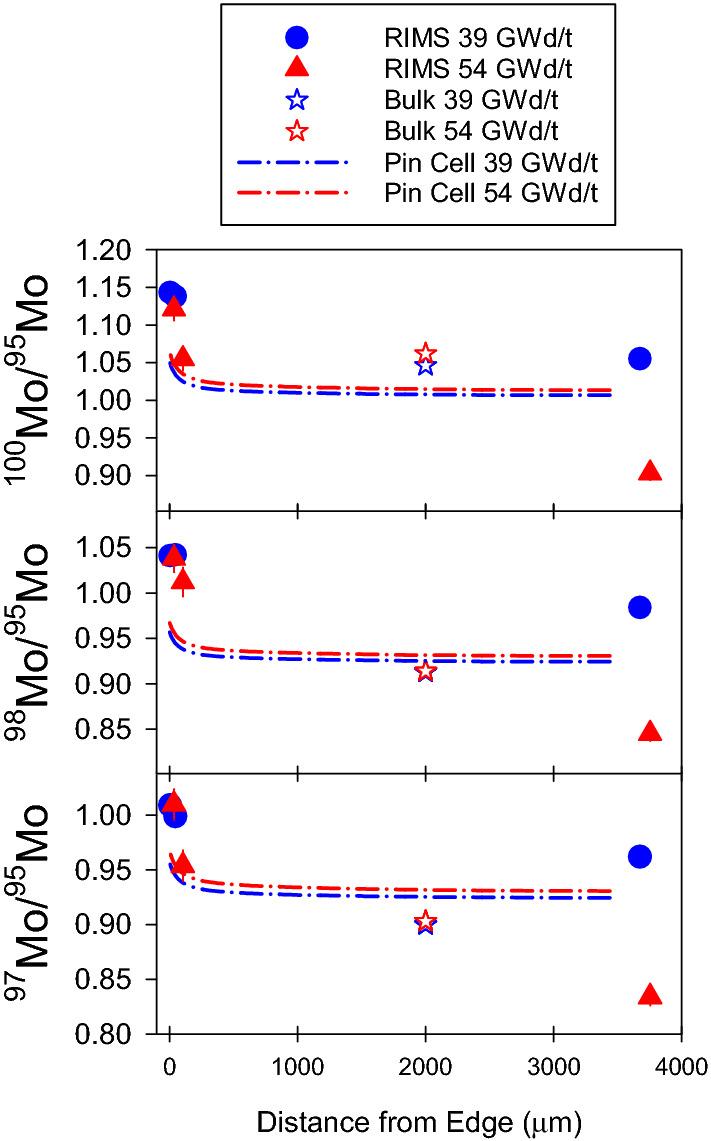
Mo isotope ratios as a function of radial position within a fuel pellet as measured by RIMS (filled symbols) and bulk analysis (open stars), and as calculated by a pin cell model (dash-dot lines). 1σ error bars are included but are generally smaller than the data symbols. Bulk analyses are arbitrarily set to a position of 2000 μm from the pellet edge.

Table 3 gives the Ru and Mo isotopic compositions measured in each sample. Figure 4 shows measured Mo isotope ratios, along with bulk analysis values and pin cell model predictions. The pin cell model provides elemental and isotopic compositions as a function of radial position within a fuel pellet. The data and models are in broad agreement for Mo: the bulk analyses and pin cell models agree within 5%. The RIMS measurements and pin cell models show the skin effect, which was also seen in actinide isotope compositions in these and similar samples^6,8^. The model agrees with the measured values within ~ 10%, however the model predictions and bulk analyses show very little difference between the two pellets, whereas the RIMS measurements show significant differences, particularly in the pellet centers.

**Table 3:** Isotope ratios measured in spent fuel samples.

Sample	Burnup (GWd/t)	Position (μm)	^90^Sr/^88^Sr	^97^Mo/^95^Mo	^98^Mo/^95^Mo	^100^Mo/^95^Mo	^100^Ru/^101^Ru	^102^Ru/^101^Ru	^104^Ru/^101^Ru
A	39	3755	0.581(6)	0.962(8)	0.984(7)	1.055(8)	0.0727(12)	0.932(7)	0.486(4)
B	39	105	0.584(9)	0.999(10)	1.042(9)	1.138(11)	0.0827(17)	0.960(9)	0.618(6)
C	39	35	0.566(7)	1.009(10)	1.041(10)	1.143(11) 14	0.0739(16)	0.978(9)	0.653(7)
D	54	3675	0.572(7)	0.834(8)	0.845(8)	0.903(9)	0.0376(9)	0.930(8)	0.543(5)
E	54	45	0.575(7)	0.954(14)	1.012(16)	1.055(16)	0.0955(19)	0.981(9)	0.607(6)
F	54	5	0.577(6)	1.010(14)	1.038(15)	1.121(15)	0.0969(18)	0.996(9)	0.671(7)
MSWD			0.8	55	72	85	328	11.5	174

Uncertainties are 1σ (see Supplemental Material for calculation of uncertainty). Sample positions are given as distance from the edge of the pellet.

MSWD: mean square weighted deviation.

**Figure 5:**
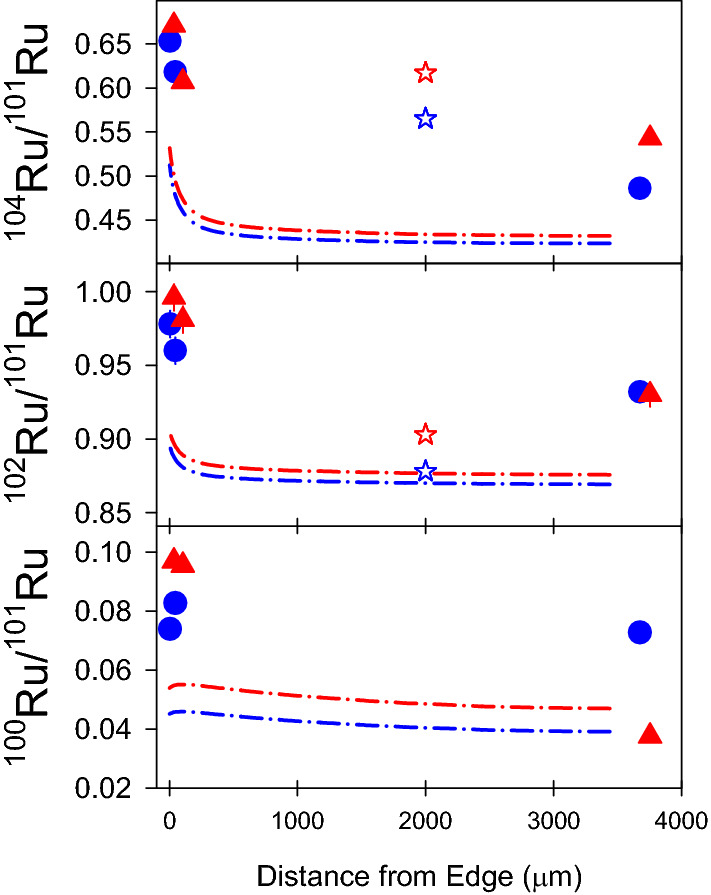
Ru isotope ratios as a function of radial position within a fuel pellet. See Fig. 4 caption and legend for details.

Figure 5 plots the Ru isotopes. The skin effect is evident in both the RIMS measurements and pin cell models, however there is considerable disagreement between the three data sets. The RIMS and bulk analyses agree for ^104^Ru/^101^Ru but disagree significantly with the model. The bulk ^102^Ru/^101^Ru ratios agree well with the model but are significantly different from the RIMS values. Figure 5 also shows the ^100^Ru/^101^Ru ratio, for which there is no bulk analysis. Here the pin cell model predicts essentially no skin effect, but one of the pellets shows a dramatic increase in ^100^Ru/^101^Ru at the edge while the other does not.

The Mo and Ru isotopic compositions are expected to vary with position within the reactor primarily due to variations in burnup and *F*_*Pu*_. Burnup and *F*_*Pu*_ are only loosely correlated. Burnup is related to the total number fissions, which produces both fission products and neutrons. Fission product elemental and isotopic compositions are modified by neutron capture during reactor operation. Neutron irradiation also generates ^239^Pu via capture on ^238^U, which increases *F*_*Pu*._ In the case of Mo and especially Ru, the isotopic composition of these fission products is strongly affected by whether the fissioning isotope is ^235^U or ^239^Pu. However, the amount of ^239^Pu produced, and hence *F*_*Pu*_, depends strongly on the character of the local neutron spectrum, which varies between the center and edge of a fuel pellet. Our pin cell models predict that burnup increases by a factor of about 1.3 from center to edge, but ^239^Pu concentration increases by a factor of 3. The same 3 × increase in ^239^Pu at the pellet edge has been measured in irradiated PWR fuel including some from samples taken from the same axial position as this work^5,7,8^. This is due to the skin effect, i.e., the strong capture of some epithermal neutrons near the edge due to resonances in the capture cross section of ^238^U. These epithermal neutrons are then greatly reduced in the pellet center due to the resonance self-shielding effect. This leads to higher *F*_*Pu*_ near the pellet edge that is out of proportion to the local number of total fissions, therefore burnup alone is not sufficient to predict local fission product compositions.

**Figure 6:**
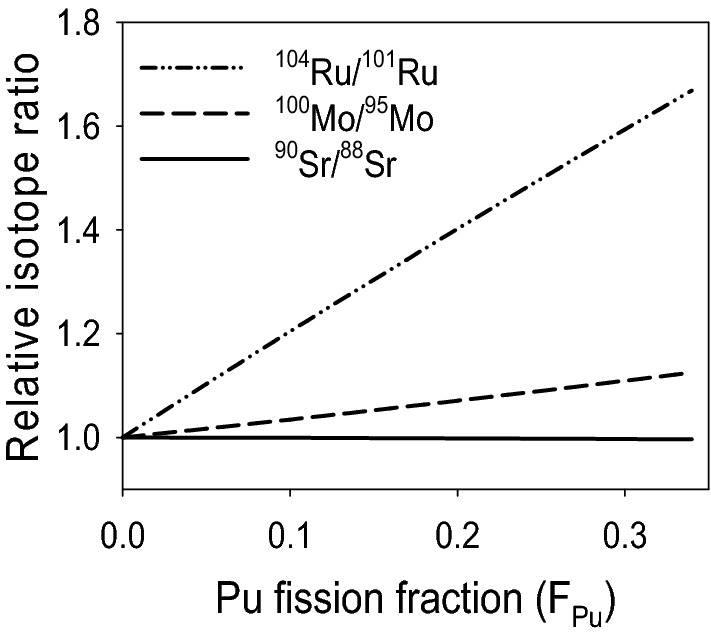
Expected change in selected Sr, Mo, and Ru isotope ratios due solely to fission yield as a function of Pu fission fraction (*F*_*Pu*_). Ratios are normalized to their values at *F*_*Pu*_ = 0.

The effect of *F*_*Pu*_ is in some sense isolable, i.e. one can understand how isotope ratios change with *F*_*Pu*_, and explain why Ru has been proposed as an indicator of the relative ^239^Pu fissions^10^. Figure 6 shows the expected change in the ^100^Mo/^95^Mo and ^104^Ru/^101^Ru ratios as a function of *F*_*Pu*_ alone (i.e., ignoring neutron capture on Mo and Ru). Here we normalize by the light fission product isotopes of Mo and Ru as these ratios should respond most strongly to changes in *F*_*Pu.*_ In the figure, the ratios are normalized to their values at *F*_*Pu*_ = 0. The ^101^Ru/^104^Ru ratio increases by > 60% as *F*_*Pu*_ rises from 0 to 0.35, reflecting the strong differences in the ^235^U and ^239^Pu fission yields of ^101^Ru and ^104^Ru. The measured ^101^Ru/^104^Ru and ^100^Mo/^95^Mo ratios do increase as expected at the pellet edge as seen in Figs. 4 and 5.

In addition, fission produces neutrons for capture on fission products, which changes isotope ratios as capture cross sections can vary widely among isotopes. For example, the thermal neutron capture cross sections for ^104^Ru and ^101^Ru are 1.5 and 16.8 barns^54^, so that the ^104^Ru/^101^Ru ratio is expected to decrease with increasing burnup due to faster depletion of ^101^Ru (ignoring capture on precursor nuclei, which are all short-lived in this case). This is opposite to the expected effect from *F*_*Pu*_, in which ^104^Ru/^101^Ru increases with burnup.

These descriptions are conceptual, but can be quantified by modeling. The estimated overall *F*_*Pu*_ for our samples is ~ 0.15^51^, which corresponds to an increase in the ^101^Ru/^104^Ru ratio of ~ 30% in Fig. 6, however because *F*_*Pu*_ changes over the course of the irradiation, Ru produced early in the irradiation will have a different isotopic composition than Ru produced later, so the effect must be integrated over the irradiation history. Similarly, Ru produced early will be subject to greater neutron capture, which must also be integrated over the irradiation history. This is accounted for in the pin cell models plotted in Figs. 4 and 5. In general, the agreement between the models and the bulk data is within 5%, except for the ^101^Ru/^104^Ru ratio which differs by > 30%. However, the RIMS microprobe data, which sampled 1–2 μm regions of the 10 μm fuel samples, shows much more variability than predicted by the models or seen in the bulk measurements. Microscale variability has been seen in unirradiated UO_2_ fuel, in which the ^235^U/^238^U ratio can vary by factors of several on the micrometer scale^57–59^. While we do not know whether our samples had similar variability prior to irradiation, such large differences in the initial ^235^U enrichment from spot to spot could possibily lead to wide variations in burnup and *F*_*Pu*_, and hence in the fission product isotopic compositions, and are not accounted for in the reactor models.

The observed microscale variability of Mo and especially Ru isotopic compositions may also be influenced by the fact that these metals, along with Tc, are known to exsolve from UO_2_ solid solution to form metallic particles up to ~ 1 μm in diameter. These are known as epsilon particles because they have the ε-ruthenium crystal structure^11,12^, and are not accounted for in the reactor models. Depending on where and when this process occurs, the chemical and isotopic compositions of the particles could vary as contributions from precipitation and fission become diffusion-limited. The isotopic composition of the metals in the particles would still be subject to alteration by neutron capture, however. The probe size for the RIMS analyses was ~ 2 μm, which is smaller than the 10 μm cube and could have sampled particle-rich or particle-poor regions. This may account for the observed difference between the RIMS and bulk analyses, since epsilon particles are resistant to acid dissolution and would not be represented in the bulk analyses. For example, it may account for the behavior of the ^100^Ru/^101^Ru system, which shows a dramatic difference between the two pellets (Fig. 5). The low ^100^Ru/^101^Ru ratio observed near the center of one of the pellets may be due to a Tc-poor particle present under the probe beam during that particular analysis, so that relatively little ^100^Ru was produced after the particle formed. The unusual irradiation history of these samples makes interpretation difficult, since diffusion would have stopped and restarted many times (Fig. 1).

### Sr isotopic composition

**Figure 7:**
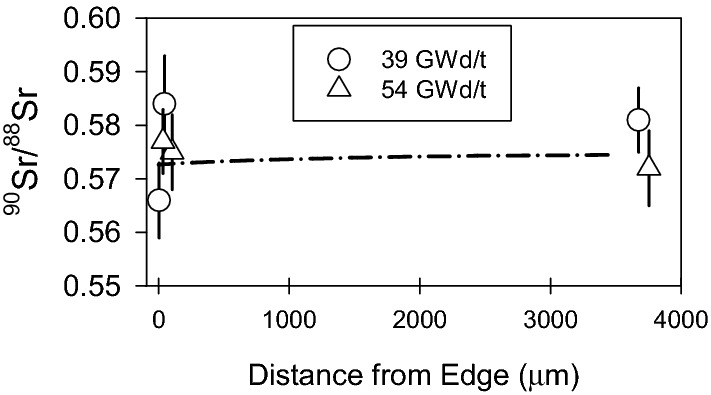
Measured ^90^Sr/^88^Sr (symbols) and pin cell model prediction (line). Uncertainties on the measured values are 1σ. The pin cell model predictions for 39 and 54 GWd/t are nearly identical so only one is shown (39 GWd/t). The pellet center is at 4000 μm, the edge is at zero.

Figure 7 shows the measured ^90^Sr/^88^Sr ratios along with the values predicted by the pin cell model as a function of the sample position within the fuel pellets. The ^90^Sr abundance from the pin cell model was decayed 40.8 years to account for the known cooling time of the samples (i.e., the time since they were discharged from the reactor). Here the model predicts essentially no change in the ^90^Sr/^88^Sr ratios as a function of the sample position or burnup. The burnups in Fig. 7 refer to the center of the pellet; the predicted burnup at the pellet edge is ~ 1.3 × higher, such that a range of burnups from 39 to 70 GWd/t are represented in the models. Even so, the pin cell model predicts no difference in the ^90^Sr/^88^Sr ratios for any of the samples, and the predictions are in excellent agreement with the measured values, which do not show the microscale variability seen in Mo and Ru.

**Figure 8:**
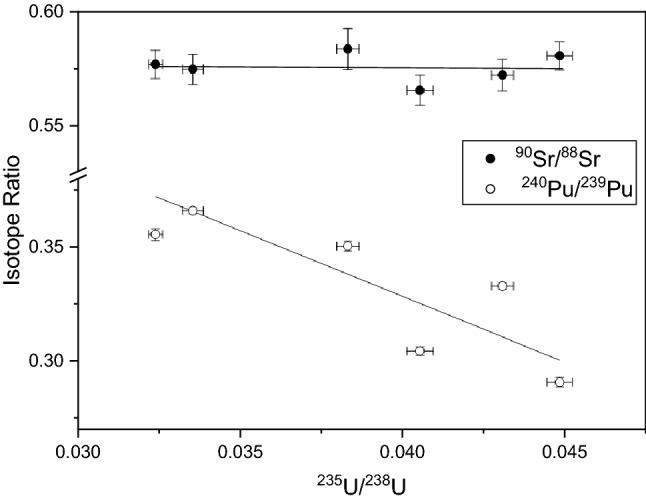
^240^Pu/^239^Pu and ^90^Sr/^88^Sr vs. ^235^U/^238^U in six samples of spent fuel. Pu and U data are from ref.^1^. Uncertainties are 1σ.

The insensitivity of the Sr ratio to burnup and skin effect is due to two main factors. First, unlike Mo and Ru, the ^90^Sr/^88^Sr ratio is insensitive to *F*_*Pu*_. The ^90^Sr/^88^Sr ratios produced by thermal neutron fission of the two isotopes differ only slightly: 1.617 for ^235^U fission vs. 1.582 for ^239^Pu fission. The change in the ^90^Sr/^88^Sr ratio over a range of *F*_*Pu*_ from 0 to 0.35 is less than 0.4%, as shown in Fig. 6. We therefore do not expect the ^90^Sr/^88^Sr ratio to show a significant skin effect. Second, the thermal neutron capture cross sections of ^88^Sr and ^90^Sr are very low, only 9 and 15 mb respectively, compared to 100–1300 mb for the Mo and Ru isotopes. We therefore do not expect neutron capture to have a significant effect, and estimates of the cooling time based on various models with and without neutron capture bear this out (below).Table 3 gives the ^90^Sr/^88^Sr ratios. The mean square weighted deviation (MSWD) of the six ^90^Sr/^88^Sr ratios is 0.8, indicating that the samples are statistically indistinguishable. This is in strong contrast to the Mo and Ru isotopic compositions, whose MSWDs range from 11 to > 300 (the upper 95% confidence limit for MSWD for six measurements is 2.56^60^. Similar scatter was seen in previous actinide measurements on these same six samples^6^. Based solely on the actinide data the samples could not be confidently assigned to the same source term, however the Sr data ties them together. Figure 8 plots ^240^Pu/^239^Pu and ^90^Sr/^88^Sr ratios as a function of ^235^U/^238^U for all six samples, using Pu and U analysis from our previous work^6^. The ^235^U/^238^U ratio serves as a rough indicator of burnup, and thus of the irradiation conditions within the reactor. The ^240^Pu/^239^Pu ratios correlate inversely with ^235^U/^238^U as expected, since higher ^235^U consumption leads to greater neutron capture on ^238^U and drives the production of Pu, which over time, leads to production of ^240^Pu via neutron capture on ^239^Pu. However, the scatter about the correlation line is considerable and is far outside the uncertainties in the measurements. In the absence of additional information, one cannot be certain that all six particles belong to the same source term. For example, even if they all are from the same reactor, they could represent more than one fueling / defueling event. The ^90^Sr/^88^Sr ratios measured in the same samples form a tight group and are independent of burnup as shown in Fig. 8. The Sr analysis establishes that all six samples belong to the same source term. In collections with particles representing multiple source terms, Sr analysis allows particles to be grouped and interpreted appropriately. However, one caveat is that Sr isotope ratios could not distinguish between samples from two different reactors if they had fuel cycles that started and stopped at the same time (within some uncertainty, in this case about half a year—see below) and were run with similar power profiles.

The independence of the ^90^Sr/^88^Sr ratio on burnup and *F*_*Pu*_ is well explained by the similarity of the ^235^U and ^239^Pu fission yields and the low neutron capture cross sections as a consequence of the closed neutron shell at *n* = 50. Barium-138 is also neutron magic (*n* = 82), and the ^137^Ba/^138^Ba ratio shows nearly the same independence in bulk measurements^61^ (here the ^137^Ba is radiogenic from ^137^Cs, which is also neutron magic). Strontium, Ba and Cs are known to form co-precipitates with metal oxides within irradiated fuel^14^. These precipitates dissolve along with the fuel meat and are represented in bulk analyses, but they may be encountered in microprobe measurements. However, strontium oxide is readily soluble in UO_2_^62^. Therefore, little fission product strontium is expected to segregate into these precipitates, and microprobe analysis should be largely unaffected by them.

### Sample age estimates

The uniform nature of Sr production across the reactor makes it a robust chronometer. A sample age can be calculated from the measured ^90^Sr/^88^Sr ratio in spent fuel according to:

1 $$t=\frac{-\mathrm{ln}\left(\frac{R}{{R}_{0}}\right)}{\lambda }$$

where *t* is the age, *R* is the measured ^90^Sr/^88^Sr ratio at time *t*, *R*_*0*_ is the initial ratio, and *λ* is the ^90^Sr decay constant (0.02407 year^-1^). Unlike traditional parent/daughter chronometers such as ^241^Pu/^241^Am, the initial ^90^Sr/^88^Sr ratio must be known to calculate an age. Simply using the relative thermal fission yields of ^90^Sr and ^88^Sr as an estimate of *R*_*0*_ gives an age consistent with the irradiation period, as shown below. An estimate of the cooling time can also be obtained setting *R*_*0*_ to the ^90^Sr/^88^Sr ratio at discharge calculated by reactor modeling.

**Figure 9:**
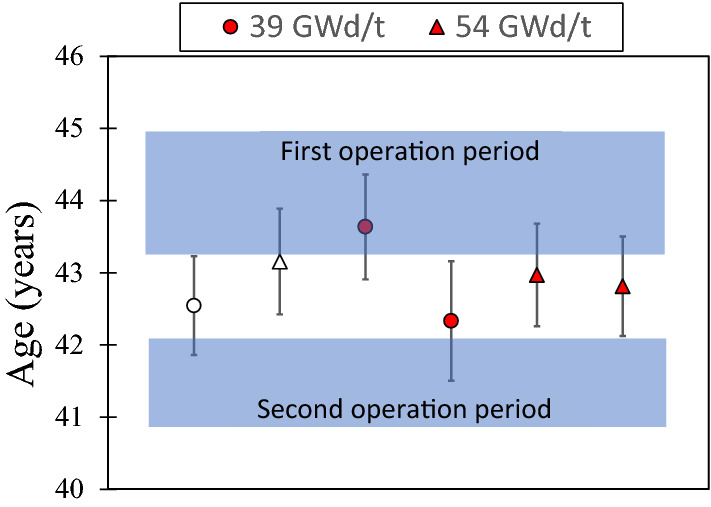
Sample ages determined using ^235^U thermal fission yields for ^90^Sr and ^88^Sr to calculate the initial ^90^Sr/^88^Sr ratio. Blue bands represent the two periods of operation of the reactor (See Fig. 1). Open symbols correspond to samples from pellet centers; filled symbols from pellet edges. Uncertainties are 1σ.

Figure 9 shows calculated sample ages compared to the known operation period of the reactor. We used ^235^U thermal fission yields from ENDF/B-VIII^54^ to estimate *R*_*0*_ in Eq. (1) as 1.618(19). This method requires no assumptions or reactor models, only the tabulated fission yields and ^90^Sr half-life. There is no significant difference in the calculated ages of samples from pellet centers or edges, as expected from the statistical indistinguishability of the measured Sr ratios. The difference in calculated ages of the 39 GWd/t and 54 GWd/t pellets was only 0.1 years, against 1σ errors of 0.7–0.8 year for individual measurements. For irradiation times short compared to the half-life of ^90^Sr, the age determined this way should correspond to some time during the irradiation period. The mean calculated age for the six samples is 42.9(6) year, which lies exactly midway between the irradiation start and stop (45 and 40.8 year respectively). Ages derived from ^241^Pu-^241^Am chronometry on similar 10–20 μm samples from the same reactor are also consistent with the known age^8^, however they have much larger analytical uncertainties and scatter than the ^90^Sr ages.

**Figure 10:**
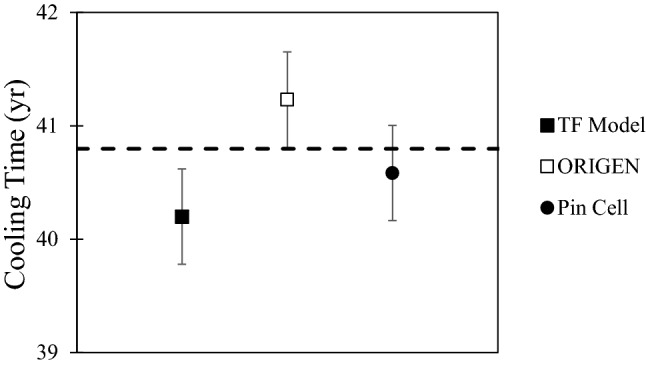
Cooling times calculated from various reactor models. The known cooling time of 40.8 years is indicated by the dashed line. Uncertainties are 1σ.

In general, the sample age calculated using the fission yields alone corresponds to the cooling time plus roughly half of the irradiation period. In this case, since we know the reactor history and cooling time, we used reactor modeling to estimate the ^90^Sr/^88^Sr ratio at discharge, which was then used in Eq. (1) to calculate the cooling time irrespective of the irradiation time. This allows us to compare models. The ^90^Sr/^88^Sr ratio changes due to both production and decay during the irradiation, and all three models showed it decreasing by 10% during the 1550-day irradiation period as burnup proceeds. For comparison, decay alone would change the ratio by only 6.6% over the same period. Figure 10 shows the results for the ORIGEN, pin cell, and TF models. Here the ^90^Sr/^88^Sr ratios of the six samples were averaged to give a single age estimate for each model. All three model ages are in excellent agreement with the known cooling time of 40.8 year. In particular, the simple TF model is in agreement with the more sophisticated models.

The agreement between the TF and more rigorous models validates the assumption that neutron capture on Sr—which is accounted for in the ORIGEN and pin cell models but omitted in the TF model—is negligible. The TF model is also robust with respect to *F*_*Pu*_, which is implicit in the other models but is adjustable the TF model. Changing *F*_*Pu*_ from zero to 0.25 changes the cooling time estimate by only 0.1 year. We used a constant *F*_*Pu*_ of 0.15, which is consistent with the fraction determined from bulk analysis^51^. The TF method is also robust with respect to the details of the power history of the reactor. The actual power experienced by these samples was far from constant and quite atypical for a PWR power reactor, as shown by Fig. 1. However, running the TF model at a constant power for each of the two irradiation periods changed the cooling time estimate by only 0.35 year.

## Conclusions

RIMS analysis of spent nuclear fuel consumes very little material and gives isotopic information on a variety of elements. The methods described here for fission products and elsewhere for actinides^6^ measure the isotopic compositions of three elements simultaneously, thereby further reducing material consumption. The Mo and Ru isotopic compositions show the skin effect but are not always in agreement with bulk measurements or models, possibly due to exsolution from the UO_2_ matrix and formation of epsilon particles, which makes interpretation difficult.

RIMS allows direct measurement of ^90^Sr without isobaric interference from the daughter product ^90^Zr, and provides a means to correct for contamination with non-fission Sr. Because the ^90^Sr/^88^Sr ratio is insensitive to both *F*_*Pu*_ and neutron capture, it does not vary as a function of sample position or burnup. This is in strong contrast to the isotopic compositions of actinides and other fission products measured in these same samples, which showed considerable variation. As a result, Sr analysis can be used to group samples by source term (i.e. reactor cycle for a given reactor), which can otherwise be difficult. Further, ^90^Sr/^88^Sr serves as a robust chronometer and is easily measured with precision sufficient to constrain the midpoint of the fuel irradiation accurately to within 0.6 year (1σ), even with the unusual power history experienced by these samples. Cooling time can be estimated by knowing the ^90^Sr/^88^Sr ratio at discharge, which is obtained from reactor modeling. The models explored here span a range from a very simple fission-only model to a relatively complicated spatially resolved pin cell model, and all yield cooling times in excellent agreement with the known value of 40.8 year.

## Supplementary Information

Supplementary Information.

## Data Availability

The datasets used and/or analyzed during the current study and supporting the conclusions of this article are included in this article. These datasets are also available from the corresponding author upon reasonable request.
