# Supplementary material for: Simultaneous isotopic analysis of fission product Sr, Mo, and Ru in spent nuclear fuel particles by resonance ionization mass spectrometry
Source: Sci Rep. 2023 Mar 30;13:5193. doi: 10.1038/s41598-023-32203-5 (PMC10063544; doi:10.1038/s41598-023-32203-5)
Supplement: Supplementary file 1 — Supplementary Information. [file 41598_2023_32203_MOESM1_ESM.docx]

## **Supplementary Information**

**Normalization by standards**

Raw isotope ratios were corrected for instrumental fractionation by normalizing to a standard:

$R=\frac{R_{sample}}{\left( \frac{R_{std}}{R_{known}} \right)}$ (S1)

where *R* is the corrected isotope ratio, *R_sample_* is the ratio measured in the sample, *R_std_* is the ratio measured in the standard, and *R_known_* is the known isotope ratio of the standard.

**Uncertainty in isotope ratios**

The uncertainties in isotope ratio measurements are calculated from counting statistics:

$\sigma=R\sqrt{\left( \frac{1}{N_{1}}+\frac{1}{N_{2}} \right)_{sample}+\left( \frac{1}{N_{1}}+\frac{1}{N_{2}} \right)_{std}}$ (S2)

where *N_i_* is the number of counts of a given isotope in either the sample or the standard. In practice the number of counts in the standard is much higher than in the sample, so the uncertainty is dominated by the number of counts in the sample.

**Uncertainty in sample age**

The uncertainty in the age of a sample is constant for a given relative standard deviation (RSD), *μ*, of the measured ^88^Sr/^90^Sr ratio, regardless of the age of the sample. This follows from the relation between the age and the ^90^Sr/^88^Sr ratio:

$t=\frac{-\ln\left( \frac{R}{R_{0}} \right)}{\lambda}$ (S3)

where *t* is the elapsed time (i.e. time since *R=R_0_*), *R* is the measured ^90^Sr/^88^Sr ratio at time *t*, *R_0_* is the ratio at *t* = 0, and *λ* is the decay constant. The uncertainty in *t* is then given by

${\sigma_{t}}^{2}=\left( \frac{\partial t}{\partial R} \right)^{2}{\sigma_{R}}^{2}+\left( \frac{\partial t}{\partial R_{0}} \right)^{2}{\sigma_{R_{0}}}^{2}+\left( \frac{\partial t}{\partial\lambda} \right)^{2}{\sigma_{\lambda}}^{2}$ (S4)

The uncertainty in *λ* is vanishingly small in this context and can be ignored (including the uncertainty in λ adds two days to the overall uncertainty for a sample aged 40 years). Applying Equation S4 to Equation 3 and noting that $\sigma_{R}=\mu_{R}R$ and $\sigma_{R_{0}}=\mu_{R_{0}}R_{0}$, gives (after simplification):

$\sigma_{t}=\frac{1}{\lambda}\sqrt{{\mu_{R}}^{2}+{\mu_{R_{0}}}^{2}}$ (S5)

Equation S5 shows that the uncertainty in the age is given by the RSDs of *R* and *R_0_*. The RSD on *R_0_* comes from the uncertainties in the fission product yields:

$\mu_{R_{0}}=\sqrt{\left( \frac{\sigma_{88}}{Y_{88}} \right)^{2}+\left( \frac{\sigma_{90}}{Y_{90}} \right)^{2}}$ (S6)

where *Y* and *σ* are the fission yields and their uncertainties. Using the data from ENDF/B-VIII.0, gives $\mu_{R_{0}}$ = 1.1%. Thus for a ^90^Sr/^88^Sr measurement uncertainty of 1%, the uncertainty in *t* is 0.62 yr (~8 months) regardless of the age of the sample.
